# Prescription drug use during pregnancy in Southern Tigray region, North Ethiopia

**DOI:** 10.1186/s12884-017-1359-8

**Published:** 2017-06-05

**Authors:** Fantahun Molla, Admassu Assen, Solomon Abrha, Birhanetensay Masresha, Arega Gashaw, Abrham Wondimu, Yared Belete, Wondim Melkam

**Affiliations:** 1 Pharmaceutics Department, School of Pharmacy, College of Health Sciences, Mekelle University, Mek’ele, Ethiopia; 2 Department of Pharmacy, College of Medicine and Health Sciences, Wollo University, Dessie, Ethiopia; 3 Pharmacology and Toxicology Department, School of Pharmacy, College of Health Sciences, Mekelle University, Mek’ele, Ethiopia; 4 Clinical Pharmacy Unit, School of Pharmacy, College of Health Sciences, Mekelle University, Mek’ele, Ethiopia; 5 Social Pharmacy and Pharmacoepidemiology Unit, School of Pharmacy, College of Health Sciences, Mekelle University, Mek’ele, Ethiopia; 6 Pharmacology Department, College of Medicine & Health Sciences, Bahir Dar University, Bahir Dar, Ethiopia

**Keywords:** Pregnancy, Drug use, FDA risk classification, Tigray region

## Abstract

**Background:**

Judicious utilization of drugs rescues the fetus from the harmful effects while treating the health problems of the pregnant women. This study aimed at evaluating drug utilization pattern and its associated factors among pregnant women in Southern Tigray, Ethiopia.

**Method:**

Institution based cross-sectional study was conducted among 647 pregnant women who had been attending obstetrics-gynecology and antenatal care units in different health facilities of Southern Tigray region. The study participants were selected using multistage sampling technique. Data collection was done using pre-tested semi-structured questionnaires and by reviewing antenatal follow-up cards. Descriptive and inferential statistics were analyzed, to assess drug utilization pattern and its associated factors among pregnant women, using SPSS version 20 software.

**Results:**

Of 647 pregnant women, 87.5% were prescribed with at least one medication. As per the United States Food and Drug Administration (US-FDA) risk classification system, 87.7, 7.9, 3.9, and 0.5% of the prescribed drug were from category A, B, C and D, respectively. Prescription drug use was more likely among gynecology ward visitors [AOR = 8.97, 95% Cl (2.69–29.88)] and among those who visited health facilities for the first time during their first [AOR =2.65, 95% Cl (1.44–4.84)] and second [AOR = 2.50, 95% Cl (1.36–4.61)] trimesters.

**Conclusion:**

Majority of the study population used safe and appropriate medications according to US-FDA risk classification system, with the exception of low proportion (0.5%) of medication with potential risk for the fetus. The average number of drug prescribed per pregnant women was in the recommended range of WHO drug use indicators guideline.

## Background

Ever since the incidence of thalidomide disaster, utilization of medication during pregnancy has become a great concern in health care systems due to the potential fetal risk [1, 2]. However, the reference to avoid all drugs during pregnancy is unrealistic and may endanger both the mother and fetus from complications of untreated acute and chronic medical disorders [3, 4]. Despite its own limitation, judicious selection of drugs based on the United States Food and Drug Administration (FDA) category which classifies drugs into five main categories: A, B, C, D, and X, is crucial to curb the risk of prescribed drugs to the fetus [5, 6].

So far, a significant number of studies have been conducted in various developed [7, 8] and developing countries [2, 6, 9–11]. Most of them reported the prescription and consumption of surprisingly a large number of drugs by pregnant women with substantial proportion of drugs from category D and X of US-FDA risk classification system.

In Ethiopia, a few studies have been conducted and reported the use of unsafe medications during pregnancy. Studies in Addis Ababa and Bahir Dar revealed that 71.3 and 88.4% of the pregnant women used at least one prescription drug of which about 4 and 11% of them received drugs from category D or X of the US FDA risk classification, respectively [6, 12].

From the preceding studies, there was an excessive consumption of medication by the pregnant women and a considerable number of them were from category D and X. There is, therefore, a need to know drug utilization trends and associated risk factors among these segments of the community in our country in general and in the region in particular. Moreover, no data is available on the current status of drug utilization pattern among pregnant women in southern Tigray region which the current study aimed to generate.

## Methods

### Study area and design

An institution based cross-sectional study was conducted, in February 2015, among pregnant women who had been receiving services in obstetrics-gynecology and antenatal care (ANC) units in the selected health centers and hospital in Southern Tigray region. There were a total of 39 health facilities (35 health centers and 4 hospitals) in the study area. The study area is one of seven administrative zones of the Regional State of Tigray. Mekelle, the capital city of the region, is 783 km away from Addis Ababa, the capital city of Ethiopia. According to the 2007 Census conducted by the Central Statistical Agency of Ethiopia [13], the region has an estimated total population of 4,314,456, of which 2,124,853 were men and 2,189,603 were women; urban inhabitants account 842,723 or 19.53% of the population.

### Sample size determination and sampling procedure

A single population proportion formula was used to calculate the sample size. Using the 41% prevalence of drug exposure of pregnant women in Mekelle city [14], 5% margin of error at 95% confidence level, a design effect of 2, and 10% inclusion of non-response rate, the calculated final sample size was 709. Multistage sampling technique was employed to select the health facilities. There were 35 health centers and 4 hospitals in the study area. In stage I, 20% from each stratum (health center and hospital) was included in the study to ensure the representativeness of the sample. Accordingly, six health centers (Adishehu, Betemera, Korem, kukuftu, Alamata, Timuga) and one hospital (Michew hospital) were selected by lottery method. In stage II, the number of study participants from each study site was determined using the proportion to population size (probability proportional to size) method and in stage III the samples determined in stage II were allocated to each selected health facilities using simple random sampling.

### Data collection and analysis

Data were collected using pre-tested semi-structured questionnaires and by reviewing antenatal follow-up cards of pregnant women. The semi-structured questionnaire was employed to collect socio-demographic data, obstetric and medical history of pregnant women. The ANC chart was reviewed to evaluate drugs which had been prescribed previously. The data extraction forms were used to collect information on the total number of ANC visits, gestational age, and drugs prescribed during each trimester. Data on medication were not collected for the entire pregnancy but for the trimesters at which the pregnant women were at the time of data collection. Data were collected by experienced nurses who had been working in ANC rooms.

Quantitative data were cleaned and entered using EPI-INFO version 3.1.5 and analyzed by SPSS version 20 software. Contingency tables were created to assess frequencies, and percentages of the variables were done in order to describe them in relation to the study population. The odds ratio was used to assess the association between dependent and independent variables. Variables which showed a statistically significant association (*P* < 0.05) in bivariate analysis were considered for multivariate model.

### Ethical considerations

Ethical approval and clearance were obtained from the Ethical Review Committees of both the College of Health Sciences of Mekelle University and Tigray Regional Health Bureau. At all levels, officials were contacted and permission was secured. The purpose of the study was explained to the study participants, confidentiality was ensured and verbal consent was obtained before data collection.

## Results

Of the total 709 questionnaires distributed to be filled by the data collectors, 647 were filled completely which gives a response rate of 91.3%. The remaining 62(8.7%) questionnaires were excluded from the analysis for gross incompleteness and inconsistency of responses.

### Socio-demographic characteristics

Socio- demographic characteristics of pregnant women in Southern Tigray region is presented in Table 1. Most of the pregnant women (59.2%) were in the age group of 18–25 years with the mean age of 24.88 ± 5.17 years. Majority of the study participants were Orthodox Christian (80.4%) and married (95.8%). Regarding residency, more than half of them (57.8%) were urban dwellers, and 46% of them attended formal education.

**Table 1:** Socio-demographic characteristics of pregnant women in Southern Tigray region, Ethiopia, February 2015 (*n* = 647)

Variable	Frequency (%)	Percentage (%)
Age group		
18–25	383	59.2
26–30	201	31.1
31–35	37	5.7
36–40	26	4.0
Marital status		
Single	16	2.5
Married	620	95.8
Divorced	11	1.7
Educational status		
Illiterate	345	53.3
Primary education	163	25.2
Secondary education	98	15.1
Tertiary education	41	6.3
Employment status		
Employed	111	17.2
Unemployed	536	82.8
Residency		
Urban	374	57.8
Rural	273	42.2

### Obstetrics and medical information

Most of the pregnant women (84.7%) visited the health facilities to attend ANC service and the remaining visited obstetrics- gynecology ward (Table 2). Concerning the time of their first visit,34.2, 35.2 and 30.6% pregnant women visited the health facilities for the first time during the first, second and third trimesters of their current pregnancy, respectively. Majority (71.9%) of the women visited the service centers for a maximum of 2 times (1–2 times). The main reason for their visit was seeking ANC services (96.9%). Over half (61.5%) of the pregnant women were multigravida, and 88.9% of them were planned pregnancies. Apart from 2.3% HIV positive pregnant women, none of the respondents reported any kind of chronic disease. Among 440 pregnant women who had hemoglobin test, 4.1% were anemic. Only three women had a history of hospitalization during their current pregnancy. Forty-seven respondents had a history of either spontaneous (80.9%) or induced (19.1%) abortion.

**Table 2:** Pregnancy-related variables of respondents in Southern Tigray region, Ethiopia, February 2015 (*n* = 647)

Variable	Frequency	Percentage (%)
Gravidity		
Primigravida	249	38.5
Multigravida	398	61.5
Pregnancy status		
Planned	575	88.9
Mistimed	53	8.2
Unwanted	19	2.9
Number of visit to health facilities		
1–2 times	465	71.9
3–4 times	178	27.5
≥ 5 times	4	0.6
Time of first visits of the health facilities		
First trimester	221	34.2
Second trimester	228	35.2
Third trimester	198	30.6
Reasons for visit to the health facilities		
ANC	627	96.9
Illness	19	2.9
Other	1	0.2
Visited ward		
ANC	548	84.7
Gynecology	99	15.3
Hospitalization during pregnancy		
No	644	99.5
Yes	3	0.5
Chronic illness		
No	647	97.7
Yes	15	2.3
Abortion		
No	600	92.7
Yes	47	7.3
Type for abortion		
Spontaneous abortion	38	80.9
Induced abortion	9	19.1
Hemoglobin level		
Anemic	18	4.1
Normal	422	95.9
Alcohol drinking		
No	646	99.8
Yes	1	0.2
Smoking		
No	646	99.8
Yes	1	0.2
Khat use		
No	637	98.5
Yes	10	1.5

### Drug use characteristics

A total of 566 (87.5%) pregnant women were prescribed with 634 drugs including supplements, (Table 3). The average number of drug prescribed per pregnant women was 1.1. Almost all (98.2%) of those prescribed with drugs had received supplemental drugs. Iron/folic acid (95%) was the most commonly prescribed of supplements; the remaining 5% was multivitamins.

**Table 3:** Medications used by pregnant women at different trimester and their US FDA risk classification in Southern Tigray region, Ethiopia, February 2015 (*n* = 634)

Prescribed drugs	1st trimester	2nd trimester	3rd trimester	Total	Drug class	FDA class
Iron/folate	221	228	87	536	Supplements	A
Multivitamins	7	10	12	29	Supplements	A
Amoxicillin	2	12	10	24	Antibiotics	B
Metronidazole	-	-	1	1	Antiprotozoal	B
Clotrimazole	-	-	2	2	Antifungal	B
Antacid	3	2	4	9	Drug for PUD*	C
Paracetamol	2	6	7	15	Analgesic	B
Cotrimoxazole	-	-	1	1	Antibiotics	D
TDF/3TC/EFV	8	6	1	15	Antiviral	C
Cephalexin	2	1	2	5	Antibiotics	B
Diclofenac	1	-	2	3	Analgesic	C/D
Ceftriaxone	-	1	-	1	Antibiotics	B
Metoclopramide	1	-	-	1	Antiemetic	B
Benzathine penicillin	1	-	-	1	Antibiotics	B

*PUD- Peptic Ulcer Disease

Of the total 634 prescribed drugs, 78 (12.3%) were non-supplemental drugs: antibacterial, antiviral, antifungal, analgesics, antiemetic and others; the dominant prescription being antibiotics followed by analgesics (Table 3). More than two third of the non-supplemental drugs were prescribed during the second (36%) and third (38.5%) trimesters.

According to the FDA pregnancy risk category for prescription drugs, the present study showed that 87.7, 7.9, 3.9, and 0.5% of the drugs belongs to risk classification A, B, C and D, respectively.

### Factors associated with pregnant women’s exposure to prescribed drugs

From the bivariate regression analysis, the type of ward, numbers of visits to the health facilities, trimester at the first visit, and gravidity were significantly associated with exposure to prescribed drugs (Table 4). These independent variables were further considered for multivariate analysis. Accordingly, multigravida pregnant women were less likely to be prescribed with the drugs as compared to primigravida [AOR = 0.43, 95% Cl (0.24–0.78)]. Similarly, those pregnant women with a 3–4 times visit had less chance of being exposed to drugs in comparison to those pregnant women who had 1–2 times visit [AOR = 0.50, 95% Cl (0.29–0.85)]. However, those who sought medical help in gynecology ward were found to be 9 times more likely to have a prescribed drug as compared to those who visited ANC ward [AOR = 8.97, 95% Cl (2.69–29.88)]. Pregnant women who visited the wards for the first time during the first [AOR =2.65, 95% Cl (1.44–4.84)] and second [AOR = 2.50, 95% Cl (1.36–4.61)] trimesters were with high odds of being prescribed with drugs.

**Table 4:** Factors associated with exposure to prescribed drugs among pregnant women in Southern Tigray region, Ethiopia, February 2015

Variable	COR (95% CI)	AOR (95% CI)	*P*- value
Gravidity			
Primigravida	1		
Multigravida	0.38 (0.21–0.67)	0.43 (0.24–0.78)	0.006
Number of visits to health facilities			
1–2 times	1		
3–4 times	0.40(0.24–0.65)	0.50(0.29–0.85)	0.011
≥ 5 times	0.10(0.01–0.74)	0.15(0.01–1.33)	0. 090
Ward			
ANC	1		
Gynecology	5.31(1.64–17.17)	8.97(2.69–29.88)	0.001
First visit to the health facility			
First trimester	2.62(1.47–4.65)	2.64(1.44–4.84)	0.002
Second trimester	2.71(1.53–4.81)	2.50(1.36–4.61)	0.003
Third trimester	1		

COD: crude odds ratio; AOD: Adjusted odds ratio

## Discussion

Prescribing drugs during pregnancy presents a challenge to physicians as pregnant women must be treated while protecting the fetuses against possible side effects of the drugs [15]. This would in turn be dependent on the proportion of drugs received by pregnant women during the course of their gestational period. The prevalence of drug utilization during pregnancy in this study was found to be 87.5%. This value is higher as opposed to the study conducted in United States, Italy and Ethiopia that reported 82, 75 and 71.3%, respectively [7, 12, 16], but comparable with the report of WHO which is 86% [17]. The observed higher proportion of the drug utilization in this study could be partly attributed to the supplements prescribed as 98.2% of the pregnant women who were prescribed with medication received supplements in the current study. From the supplement categories, iron/folic acid (95%) was the most frequently received supplement in the present study which is an appealing finding when it is compared to a study conducted in eight rural districts of Ethiopia where only 35.4% of the women who gave birth in the preceding year were prescribed with iron tablets [18].

The most frequently prescribed non-supplemental drugs were antibiotics (41%) followed by analgesics (23%) which is in agreement with the previous studies done in Ethiopia [19, 20]. Antibiotics are one of the most frequently prescribed medications during pregnancy with estimated range of 19 to 44% [21]. Paracetamol was the most widely used analgesic and antipyretic drug in this study and higher utilization was observed in the third trimester.

The US-FDA pregnancy risk classification system was used to evaluate the risk levels of drugs prescribed during pregnancy. The majority of prescribed drugs in the present study were safe as 87.7 and 7.9% were found to be category A and B, respectively. Only three pregnant women (0.5%) were prescribed from category D drugs in the current study, which is by far lower as compared to the findings of the other studies [6, 19, 20]. However, even a small increase in the risk of birth defects may have significant implications for public health. This result emphasizes the need for thorough consideration while prescribing drugs to pregnant women. The drugs which were prescribed from category D were diclofenac (when prescribed at third trimester) and cotrimoxazole. As opposed to different studies [6, 19, 20] conducted in other parts of Ethiopia, where 0.4–11% of pregnant women was prescribed with drugs in category X, there was not a single pregnant woman who was prescribed with category X drugs in our study.

Multigravida pregnant women were less likely to be prescribed with drugs as compared to primigravida in the current study. The reason might be multigravida pregnant women would have tolerated some of the physiological changes which prompt drugs use in the case of primigravida woman. This finding is in contrast to the study conducted in Bahir Dar city Administration [6].

Those pregnant women having 3–4 times visit had less chance of being exposed to drugs in comparison to those who had 1–2 times visit. A similar trend of less drug prescription with increased number of visits to the health facilities was observed in the study conducted in Brazil [22]. This could be due to the fact that the more frequent the visiting the better is the counseling of the health providers for the pregnant women regarding the issues related to different aspects of pregnancy. This might have helped the pregnant women to manage their health problem without actually consuming drugs. In contrast, other studies reported the utilization of more drugs by the pregnant woman with more frequent prenatal visits [22, 23].

Pregnant women who had their first visit during the first and second trimester had high odds of being prescribed with drugs. This may be attributed to the fact that most of the health providers are accustomed to give supplements in the first and second trimester and the majority of drugs prescribed in this study were supplements. .

Pregnant women who sought medical help in gynecology ward were more likely to have a prescribed drug as compared to those who visited ANC ward. This finding is in accordance with the study done in Ayder referral hospital, Ethiopia, [14] where the highest average number of medications prescribed per pregnant woman was recorded in the gynecology ward.

The main limitation of this study was a temporal relationship establishment with some variables, the cross-sectional design employed in this study which could not provide much more substantial evidence of causality as compared to longitudinal design. Moreover, we did not examine over-the-counter medications and drugs dispensed in the hospital. It is possible that this could underestimate the prevalence of drug utilization particularly for drugs that are commonly available over the counter.

## Conclusions

Majority of the study population used safe and appropriate medications according to US-FDA risk classification system, with the exception of low proportion (0.5%) of medication with potential risk for the fetus. The average number of drugs prescribed per pregnant women was in the recommended range of WHO drug use indicators guideline. Even though the drug utilization pattern in this study is encouraging, the issue of prescribing drugs form category D and X should be addressed by informing the prescribers to stick to the treatment guideline. Besides, educating and counseling pregnant women and women of child bearing ages regarding the safety of drugs during pregnancy are important to reduce the risk on the fetus.
